# Neuroplasticity of the Sensorimotor Cortex during Learning

**DOI:** 10.1155/2011/310737

**Published:** 2011-09-21

**Authors:** Joseph Thachil Francis, Weiguo Song

**Affiliations:** ^1^Departments of Physiology and Pharmacology, The Robert F. Furchgott Center of Neural and Behavioral Science, and the Joint Program in Biomedical Engineering at SUNY Downstate and NUY-POLY, State University of New York (SUNY) Downstate Medical Center, 450 Clarkson Avenue, Brooklyn, NY 11203, USA; ^2^Departments of Physiology and Pharmacology, State University of New York (SUNY) Downstate Medical Center, 450 Clarkson Avenue, Brooklyn, NY 11203, USA

## Abstract

We will discuss some of the current issues in understanding plasticity in the sensorimotor (SM) cortices on the behavioral, neurophysiological, and synaptic levels. We will focus our paper on reaching and grasping movements in the rat. In addition, we will discuss our preliminary work utilizing inhibition of protein kinase M*ζ* (PKM*ζ*), which has recently been shown necessary and sufficient for the maintenance of long-term potentiation (LTP) (Ling et al., 2002). With this new knowledge and inhibitors to this system, as well as the ability to overexpress this system, we can start to directly modulate LTP and determine its influence on behavior as well as network level processing dependent at least in part due to this form of LTP. We will also briefly introduce the use of brain machine interface (BMI) paradigms to ask questions about sensorimotor plasticity and discuss current analysis techniques that may help in our understanding of neuroplasticity.

## 1. Introduction

*Neuroplasticity* refers to the ability of neurons, neural circuits, and the brain itself to be modified and to reorganize both physically and functionally. This includes, but may not be limited to, changes in the strength of synaptic connections, the formation and elimination of synapses, dendrites, and axons as well as changes in the synaptic vesicular pool and content. Recent findings have shown that the sensorimotor cortex is very dynamic and is involved not only in motor learning, but also possibly in cognitive events as well [1–6]. An obvious demonstration of the plasticity within the sensorimotor cortices are the changes in neurophysiological properties following injury, for an extensive review please see [7]. Besides injury, there is a growing body of research indicating plasticity in the sensorimotor regions that occurs at different times during sensorimotor learning, from *in vitro*, *in vivo*, to behaving animals experiments [8–11]. Plasticity can be induced via several sources, including activity-dependent, use-dependent, or persistent stimulation [9, 12–14]. 

The sensorimotor cortices appear to encode several types of movement-related parameters, ranging from muscle activation patterns to body kinematics and or dynamics to name a few [15–21]. During sensorimotor learning, the relationship between neural firing and these parameters can be changed via some form of neuroplasticity. One such change is an apparent rotation in the neurons preferred direction (PD) of movement [22, 23], which is simply the direction of hand motion that the given neuron encodes preferentially. This type of modulation has been seen not only with real reaching movements, but also is currently a topic of intense research in the BMI community [24–26]. In addition to changes in PD, there have been reported changes in the number of significantly modulated cells at different phases of adaptation and learning [27, 28] as well as changes in neural firing variability associated with increased sensorimotor performance [29]. 

Generally, sensorimotor cortical plasticity is considered to be divided into two main components [30, 31]: (1) changes in the somatotopy of sensorimotor areas induced either by injury, amputation, or learning of a skilled sensorimotor task, such as the reach, grasp, and retrieve (RGR) task for monkeys [12] and rodents [2] that may be coupled with changes to the dendritic and synaptic structures [32, 33]; (2) plasticity within the network induced by microstimulation that changes the field potentials [34]. At the behavioral level, sensorimotor learning is also divided into two components, early and late phases, the former having usually a steeper learning rate. In this paper, we will discuss mostly data provided by the rat model, but we will also include some primate work relevant to the development of BMI as a new tool for exploring sensorimotor learning.

## 2. Rat Sensorimotor Cortex and Associated Neuroplasticity

**Figure 1:**
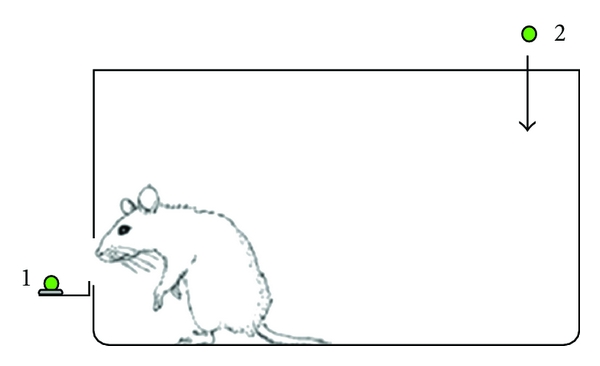
The rat reach-grasp-retrieve (RGR) task. The rat is allowed to make one controlled reach for the food pellet seen at position 1. The rat must reach through a slot that is large enough for his hand, but not his mouth or whiskers. The rat must then walk to the back of the chamber where they may receive a second food pellet. Walking to the back of the cage is necessary in order for a new food pellet to be placed at position 1.

It is not surprising that most of our knowledge pertaining to sensorimotor changes in long-term potentiation (LTP), long-term depression (LTD), morphological changes to the synaptic substrate, and in general neuroplasticity comes from rodent studies that we will now review with our focus on results from the rat reach to grasp and retrieve (RGR) paradigm. The RGR paradigm is a simple task that has been used for more than half a century to investigate sensorimotor learning, control, and performance. In short, rats are allowed to make reaching movements through a small slit that their hand and arm can fit through, but that their face and whiskers cannot (see Figure 1). It is believed that this task relies heavily on their sense of olfaction to pinpoint the target food item [35]. There are several variants of this task, but in general, the rat is allowed to make a single reaching attempt through the slot for a small food pellet that sits in a small well on a shelf at the front of the behavioral chamber. After this reaching attempt, the food pellet may be removed to encourage the rat to make controlled reaches. After the reach, the rat must walk to the back of the chamber to start a new trial at which time another food pellet is placed on the shelf at the front of the box [36]. This ensures that the rat resets its stance before making the next reaching movement. Thus, this task involves both an explicit component, which is to walk to the back to start a new trial, and implicit components, which would be the procedural sensorimotor learning, which leads to the formation of a sensorimotor memory that is expressed as an increase in performance proficiency on the task.

### 2.1. Changes in LTP, LTD, and the Synaptic Dynamic Range

Over time rats learn to increase their proficiency on this reaching task that is often tracked as a percentage of successful trials, where a success is often described as when the rat makes a single reaching attempt, grasps the food pellet, and consumes the pellet. In a series of papers published by the group lead by John Donoghue involving the work of Hess and Rioult-Pedotti as well as others, we learned that there are several changes to the level of LTP, LTD, and the overall dynamic range of synaptic plasticity in the sensorimotor cortex that are induced after learning on this reaching task. 

#### 2.1.1. GABAergic, Cholinergic, and Dopaminergic Innervation of M1 and Neuroplasticity

In one of their early papers on this subject [37], the authors demonstrated that one could induce LTP via theta burst stimulation (TBS) at the horizontal inputs to M1 in layers II/III, but this induction required the transient pre-TBS application of the GABA-A receptor antagonist bicuculline methiodide (bic). It was noted that this LTP could be enhanced if two stimulating electrodes were used simultaneously within the range of about 1 mm from the recording site within these same layers. This paper was conducted utilizing a slice preparation. In this early work, these changes in horizontal connections were viewed in light of sensorimotor cortical map plasticity that has been previously reviewed [30, 38]. In the following years, these authors described a series of experiments aimed at determining the rules of potentiation in the motor cortex of the rat [39–44]. They determined that they could also induce LTP in layers II/III by using a paired TBS protocol of the vertical pathway in conjunction with the horizontal pathway [40]. In 1999, these authors furthered our knowledge by showing that paired, or associative TBS in layer I and layers II/III, could also induce LTP without the application of pharmacological agents. They determined that this was reliant on cholinergic receptors as bath application of muscarinic blockers prevented LTP induced via paired layer I and layers II/III TBS. In fact they found that this paired stimulation in the presence of atropine caused LTD not only at the layer II/III horizontal responses, but also at layer I [44]. 

The importance of cholinergic inputs to the motor cortex on sensorimotor learning of this RGR task have more recently been shown *in vivo* as well [45, 46]. In this recent work, the researchers used injections of the immunotoxin 192-IgG-saporin (SAP) to decrease the intrinsic cholinergic inputs either globally or specifically within the motor cortices. They noted that there was a decrease in the rate of learning the RGR task when SAP was injected into the motor cortex or into the nucleus basalis/substantia innominata during the early phase of learning, which for their purposes was training days 1–4. In addition, the overall performance for the SAP groups was lower than the control groups. However, during the late phase of learning/performance, there was no significant difference between the learning rates of the SAP and control groups. These authors also noted that the animals lacking their normal cholinergic innervation did not have a learning associated caudal motor cortical map expansion as seen in the control animals. 

In addition to M1 synaptic plasticity being modulated, or gated by GABAergic and Cholinergic innervation, it has been shown that dopaminergic inputs from the ventral tegmental area also play a role in gaiting LTP induced during learning of the RGR task in the rat [47, 48]. In this work, it was noted that the dopaminergic input was only necessary for the learning phase of this task and that once the animals had learned the task, that dopaminergic depletion in M1 did not decrease performance. Along with changes in performance on the RGR task the authors showed that LTP induction in the slice preparation was altered via D1 and D2 receptor blockers, decreasing the amount of LTP that could be induced in the presence of the GABA antagonist bic, and that LTP induced after one TBS was already at the LTP limit of this preparation versus controls that could undergo even more LTP over a period of several TBS stimulations [47]. In addition to these results, they show in [47, Figure 1.A] what appears to be a significant decrease in learning/performance on the task due to a lack of noradrenergic terminals in M1, although according to the authors this did not reach significance even though all 6 data points for this group's learning curve were lower than their respective counterparts from the control group. This may indicate that this modulatory neurotransmitter system also plays some small role in gating the amount of LTP that can take place in the rat's M1.

#### 2.1.2. Behaviorally Induced Synaptic Plasticity Changes

**Figure 2:**
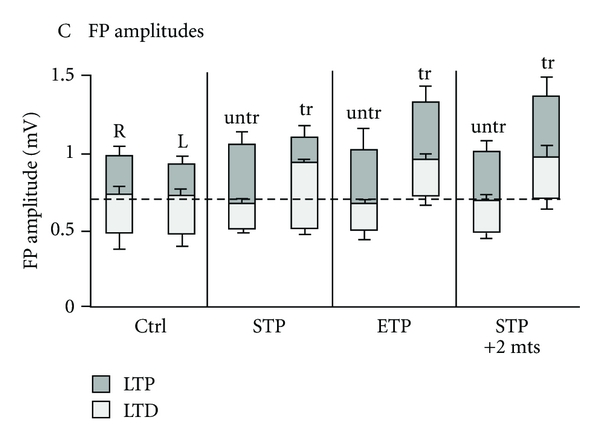
Taken from [10] with permission. Shown are the results from control animals (Ctrl), short task performance on the RGR task (STP, 3–6 days), extended task performance (ETP, 23–105 days) and short task performance followed by two months (STP + 2 mts). The trained (tr) hemisphere and the untrained (untr) hemisphere are shown for each group. The *y*-axis is the evoked field potential with LTP and LTD coded for by gray (LTP) or white (LTD). Note the change in the baseline and range after both the extended training as well as the short-task performance followed by two months.

The previous section dealt with changes in synaptic plasticity, or neuroplasticity, generally in the *in vitro* preparation and we now move onto a set of papers that proved that this type of plasticity could be induced and related to changes in performance on the RGR task [10, 34, 49]. In the first of these papers, the authors demonstrated that after learning the RGR task for 3–5 days that the evoked field potentials in M1 on the trained hemisphere, that is, the hemisphere contralateral to the arm used for the RGR task were larger than on the untrained hemisphere of the same rats [34]. Again, these field potentials were recorded from layers II/III in brain slices made from the trained animals. They also noted that the amount of LTP that could be further induced from this new baseline field potential amplitude in the trained side was less than that in the untrained hemisphere. Thus, the dynamic range of plasticity had been shifted. In essence, the trained side was near its ceiling of LTP already. This last point was then followed up on in a subsequent paper where they showed that not only was the amount of LTP inducible on the trained hemisphere near its ceiling, but the amount of LTD that could be induced was increased, and in a manner suggesting that the plasticity dynamic range stayed the same [49]. In 2007, they continued this work when they reported on how the plasticity range and its baseline would change over time with and without continued practice on the RGR task [10]. They found that the range would stay about the same over time, on the order of 23 days to months, but that the whole distribution would shift to higher field amplitudes, such that the new baseline would sit about in the middle of the plasticity range, with slightly more LTP induction possible than LTD via electrical stimulation protocols (see Figure 2). Thus, at this new point, the amount of LTP that could be induced in the trained hemisphere would increase and no longer be at its ceiling as in the earlier work (Figure 2, STP group). Likewise, the amount of LTD that could be induced would decrease as compared to the early phase changes just after learning. These changes thus keep the learning induced increase in the field potentials of the network, but the absolute amplitude of the network can now go even higher, that is further LTP can be induced. 

**Figure 3:**
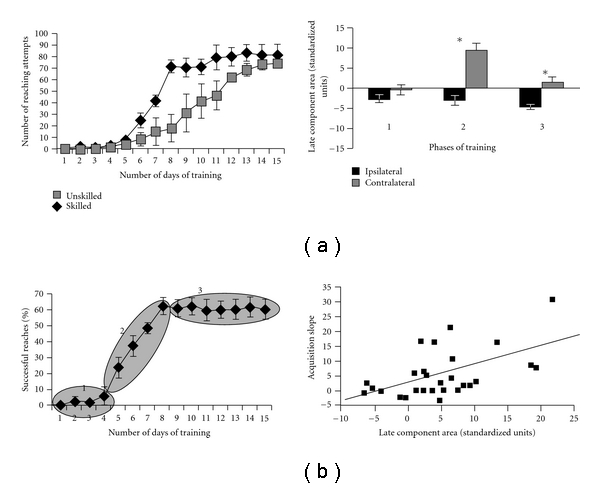
Taken from [50] with permission. The upper left panel shows the number of reaching attempts made on the RGR task over days between the skilled and the unskilled reaching groups, with the associated learning curve for the skilled group shown below it. The upper right panel shows the related changes in the late phase of the evoked field potential over the three phase of learning. Note the linear relationship between the learning curves acquisition slope on the *y*-axis of the bottom right panel and the area under the late phase field potential.

After the initial discovery that LTP was in fact associated with the increased performance on the RGR task, Monfils and Teskey tested whether the electrophysiological results would hold true in the *in vivo* setting as the previous electrophysiological studies were all *in vitro* slice preparations [50]. Previous work [51–53] had determined some of the criteria that could lead to electrophysiologically induced LTP and LTD in the neocortex in the chronic rat preparation, which has been previously reviewed [54]. Monfils and Teskey's results demonstrated that during the steep-learning curve phase there was an increase in the late component of the population response in the caudal forelimb region of M1 contralateral to the arm used. In this work, they called the initial phase of the learning curve, where there was no obvious learning and almost no reaching attempts phase one (days 1–4), phase two was the steep area of the curve where learning/(increase performance) was taking place (days 5–8), and phase three was after the rats had reached an asymptotic performance level (days 9–15) (see Figure 3). Clearly, different authors use different protocols for their rat training and the slight differences in the reaching tasks used as well as the stimulation procedures make direct comparisons slightly difficult. 

Contrary to the results from the previous *in vitro* studies the increase in field potential was only seen during the steep learning phase. They termed these changes in the field potential learning-related potentiation as opposed to LTP and LTD that they would also test via high and low frequency stimulation of the corpus collosum [50]. In agreement with the previous *in vitro* work, they noted that there were persistent differences in the amount of LTP and LTD that could be induced between the trained and nontrained hemispheres from the same rat. Specifically, the amount of LTP that could be induced was higher in the ipsilateral untrained hemisphere than the contralateral with the expected opposite effect on LTD, which is a larger induced LTD on the contralateral side compared to the ipsilateral. 

There are several differences between this Monfils and Teskey *in vivo* study and the previous *in vitro* studies [10, 34, 49] that we summarize here. First, the obvious, the *in vitro* studies used slice preparations which do not necessarily represent the system as it was before slicing, which is known to induce changes in the neural substrate. Secondly, LTP in these studies was induced in conjunction with the application of a GAGAa antagonist bic to allow LTP induction with TBS on a short timescale. All field potential test stimulation in the *in vitro* studies was done within 1 mm of the recording electrode in layers II/III, whereas in the *in vivo* work electrophysiologically induced LTP was induced over the course of days via TBS in the corpus collosum. Thus some of the differences seen between these two bodies of work could be the sites of stimulation during the test stimuli evoking the field potentials that were then used to determine LTP and LTD in addition to the aforementioned differences. With more and more labs interested in such neuroplasticity and the prevalence of awake chronic recordings in the rat we hope it is a short period of time before some comprehensive work is conducted rigorously trying to bridge the gap between these studies.

#### 2.1.3. Synaptic Changes and Map Expansion

There are several recent publications on changes that take place to the synaptic substrate during sensorimotor learning [33, 48, 55], and as previously mentioned on changes in the sensorimotor map [7, 30, 31, 56]. Thus, here we will only focus on points that relate this information to the rat RGR task and the previously mentioned results in the above sections. One of the first studies using the RGR task and demonstrating dendritic changes due to this sensorimotor learning to our knowledge was the work of [32]. Since this work, there have been many articles discussing changes in the synaptic structure induced via motor tasks other than the RGR task in the cerebellum [57] sensorimotor cortex [58], as well as after using the RGR task [2]. Kleim and his colleagues have conducted much of the work investigating changes in synaptogenesis, angiogenesis, and map plasticity utilizing the rat reaching task and other sensorimotor learning tasks [2, 58–67]. 

In essence, a summary of this work is as follows and has been previously reviewed [56, 68]. Skilled motor learning, but not simply motor activity leads to changes in the somatosensory map as well as synaptogenesis [2, 58, 60]. Skilled reaching is also associated with increases in LTP as compared to simple unskilled reaching movements [50]. In addition, motor skill acquisition is necessary for sensorimotor map changes to occur [66], and these changes occur after changes in the synaptic substrate, which in turn is preceded by changes in gene expression associated with such skill acquisition [58].

#### 2.1.4. Erasure of LTP and Its Influence

**Figure 4:**
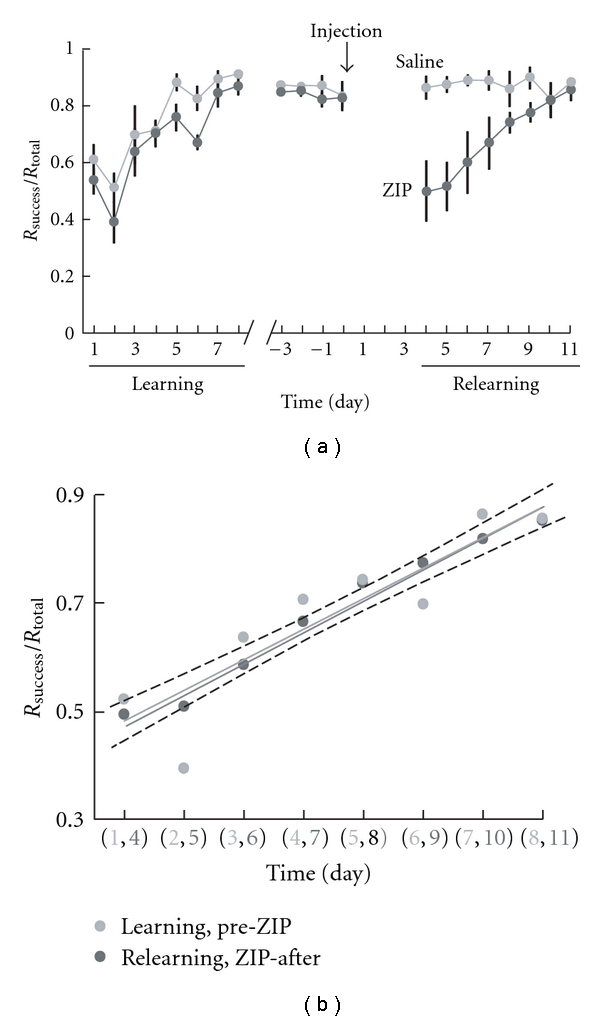
Figure and Figure legend taken and modified from [72] with permission. (a) shows the average percentage of successful reaches for both control and ZIP groups during initial learning and relearning after injection into the sensorimotor cortex. After reaching asymptotic levels of success, the animals were injected with either saline or ZIP. After 4 days rest, the animals were tested on the reaching task. Following the initial decline in performance to naive levels, the ZIP-injected rats relearned the task, and there was no significant difference between the initial learning and the relearning curves of the ZIP-injected rats (b); ANCOVA *P* = 0.80, slope; *P* = 0.35, *y* intercept). This suggests that there were no significant memory savings or damage to the cortex due to the injections, as also indicated from the lack of change in the control animals' performance after-injection. Histological analysis of brain sections indicates the spread of ZIP did not extend into subcortical regions, but encompasses several areas involved in skilled reaching including M1, M2, and S1 limb regions, see (Figure 3(b)) from [72].

Recently, a constitutively active protein kinase C isoform (M*ζ*) has been described as necessary and sufficient for the maintenance of LTP [69]. The behavioral relevance of this enzyme (PKM*ζ*) and the associated LTP has been demonstrated in a series of studies [70–72]. Using the pseudosubstrate of PKM*ζ*, zeta inhibitory peptide (ZIP), PKM*ζ*-dependent LTP was inhibited, and with it, explicit, hippocampal-dependent spatial memories [70], as well as cortically dependent taste aversion memories [71]. In our preliminary work, we bilaterally injected ZIP into the sensorimotor cortex of rats trained to an asymptotic level of success on the RGR task (Figure 4(a)) [72]. Post-ZIP injections, the rat's performance declined to prelearning baseline (Figure 4(b)). Importantly, the rats did not forget the basic structure of the task, as they continued to walk to the back of the cage in order to initiate a new trial. 

There are many open questions we are currently working on in order to increase our understanding of these preliminary observations. However, this work demonstrated that after ZIP injections, the rats performance on the RGR task declined to at least their initial naïve level, and that subsequent relearning had the same rate as the initial learning, implying no savings of the previous memory. Additionally, the results argue against significant cortical damage that would result in a slower learning rate as the damaged region would reorganize in order to regain the previous functionality, or as a different brain region would compensate for the damaged area. Based on preliminary video analysis, the difficulty the rats encountered seemed to be in the grasping, rather than reaching, implying that this is the phase of the movement which leads to the most LTP and changes in synaptogenesis. The receptive field properties in S1 may also have been disrupted and may have participated in performance decline. If so, then changes in receptive field properties may be an integral part of sensorimotor memory, further implying that learning and memory of skilled movement require plastic changes in primary sensory and motor areas. In pursuit of this idea, we will continue this work in both the rat and the monkey utilizing a combination of electrophysiological and behavioral techniques to determine the role that PKM*ζ*-dependent LTP plays in somatosensory and motor cortical processing, receptive field structure, motor map structure, synaptogenesis post-RGR learning, and performance on the RGR task.

### 2.2. The Brain Machine Interface as a New Tool for Probing Sensorimotor Plasticity

Brain-machine interfacing (BMI) is a rapidly developing field of neuroscience/biomedical engineering that may serve as a new tool to better understand neuroplasticity on a network level. The operation of closed loop BMIs depends on neural decoding from populations of neurons and on the subject's adaptation to the output of the BMI. To date, most of this feedback has come via the visual system viewing either movement of a computer courser [73–75] or of robotic actuators [76, 77]. The subject's brain then learns the relationship between its intended output movement and the subsequent real output from the BMI. We believe that just as with normal sensorimotor learning the brain is building up an internal representation, or internal model, of this relationship. Due to the fact that with a BMI the causal relationship between neural activity and actuator motions is explicitly defined by a decoder/experimenter, it is possible to directly investigate the origin and functional implications of adaptation related changes in the neural dynamics and physical substrate. Contrary to traditional methods used in motor plasticity and motor-learning research, such as *in vivo* induction of LTP and LTD, or even the RGR task, BMI technology provides a new paradigm to research synaptic and neural plasticity in awake behaving conditions with the added constraint of a defined subpopulation of the brain and a defined output transformation. 

#### 2.2.1. Plasticity during Learning to Control BMIs

As BMIs create a direct causal relationship between neural activity and actuator motions, it is possible to directly investigate the origin and functional implications of adaptation related changes in the neural dynamics. The groups lead by Andrew Schwartz and Jose Carmena have presented beautiful experiments demonstrating how the neurons modulate and reorganize after BMI learning takes place [24–27, 78–80]. In an early set of experiments, Carmena et al. demonstrated that the control of the external actuator via manual control, that is, when the monkey is using its own arm and musculature, and brain control, which is under BMI control, showed different motor learning and control strategies [78]. These authors and several other groups now have noted that the neural activity has different dynamics under brain control and manual control [27]. The preferred direction for many neurons shifted significantly between BMI control and manual control. This change in PDs may be due to a mismatch that occurs as the monkey stops moving their arms during BMI control, which means that the normal proprioceptive feedback that would be coming into these brain regions is no longer correlated with the endpoint being controlled, such as the computer courses or robotic hand. This idea was taken to action by Nicolas Hatsopoulos's team [81]. In this work, they noted an increased performance on the BMI when the arm was moved passively via an exoskeletal robotic system in an attempt to keep the proprioceptive feedback in alignment with the visual feedback of the BMI controlled courser. The robotic system used was rather compliant though and further improvements may be seen once this is taken care of and with extended practice on the system. This work is yet another example of how the BMI paradigm can be used to test certain hypothesis on the motor control system and study neural network plasticity associated with learning on such systems. 

Zacksenhouse et al. showed that during the period the animals were learning to use a BMI to control reaching movements, neurons showed both rapid and stronger plastic changes after the monkeys started operating the BMI. The enhanced modulations were not correlated with the kinematics of the movement [25, 27, 28, 82, 83]. The initial enhancement in firing rate modulations declined gradually with subsequent training in parallel with the improvement in behavioral performance. By introducing a perturbation to a BMI paradigm, Jarosiewicz et al. examined how cells in the network reorganized during a BMI learning process, and they found a functional network reorganization after BMI learning [24]. Depending on the decoder's used, such as for velocity or joint torques [21], and the BMI tasks used, plasticity in the motor cortex might occur differently [25, 82]. Conceivably, sophisticated decoding algorithms could affect or impede subsequent improvement of the BMI control in dynamic environments using intrinsic brain plasticity and adaptation. Still yet other decoders are being developed that learn themselves via reinforcement learning, which then leads to a situation where there are two learning entities, the monkey for instance and the computational agent [84]. This type of system can then be used to further study neuralplasticity due to such group learning. By fixing a linear decoder, Ganguly and Carmena further explored how the network reorganized on a timescale across multiple days [25]. They showed that the performance of the BMI can be improved with learning and significant neural plasticity in M1 was observed. Finally, a new cortical map for the neuroprosthetic representation emerged [25], which can be considered the development of an internal model of the BMI system. 

Fetz and colleagues conducted some of the earliest work along the lines of BMIs proving that given simple feedback of a neurons activity via a speaker monkeys could learn to differentially modulate the activity for reward [82, 85]. Although in those early studies they did not have the current luxuries we have that allow us to record from hundreds of electrodes simultaneously to follow the network level changes that were associated with the animals learning. Current BMI work is clearly looking into such neural plasticity these days, and it is only a mater of time before it is mixed with pharmacological studies, such as the ZIP work we presented, in order to determine the role that LTP plays within the different brain regions during such learning.

#### 2.2.2. Plasticity during Adaptation to a Locomotor BMI

Except for the instrumental conditioning of a voluntary task, BMIs could also be used in simple task such as adaption to augmenting their locomotion. BMIs were first proven to be a possibility in rats, whose motor cortical cells could be modulated to control a robot arm [86]. In order to examine neural modulation during adaptation to a BMI that was mechanically coupled to the users body, Song and Giszter presented a novel BMI using neural discharges in the hind limb region of the motor cortex in rats during locomotion to control a robot attached at the pelvis [28]. In this work, they tested how cells adapted when rats experienced (a) normal control locomotion, (b) “simple elastic loads” (a downward pelvic load produced by the robot on locomotion without any BMI neural control), and (c) “BMI with the elastic load” (in which the BMI neural control could counter this load). They found that firing rates increased in both the loaded conditions compared to baseline. Mean phases of discharging cells in the step cycle shifted significantly between BMI and the simple load condition. Furthermore, in BMI mode, over time the neural network's correlation increased. Loading alone showed none of these effects. The BMI changes in rate and correlation to force persisted or increased over repeated trials. These results show that rats have the capacity to use motor adaptation and motor learning to fairly rapidly engage hindlimb/trunk coupled BMIs in their locomotion. Motor cortical learning or adaptation may not be evoked during simpler locomotion tasks in line with previous natural locomotion studies [58].

#### 2.2.3. Plasticity during Microstimulation in Close Loop BMI

Instead of creating a direct link between firing rate and external actuators in the traditional BMI, a new close-loop BMI paradigm could also shape neural firing by electrically, or optogenetically, stimulating in sensory or motor cortical areas to provide sensory feedback for motor control. By using a similar *in vivo* cellular conditioning protocol: using spikes detected from one neuron to trigger an electrical stimulator to stimulate another neuron after proper delay, Jackson et al. demonstrated *in vivo* that neurons in motor cortex are very plastic and could be modulated following Hebb's learning rule under natural behavioral conditions [87]. In essence they formed an artificial link between one neuron that was being recorded from and a second electrode that would then be microstimulated through some short period of time (~5 msec), after a spike was recorded on the recording electrode. They found that after persistent conditioning the motor output induced via microstimulation (MiSt) at the recording electrode site shifted to the motor output caused by MiSt at the second electrode position. By using the same type of MiSt protocol as Jackson et al., Rebesco et al. examined the functional connectivity at the network level not by tracking changes in the MiSt-induced motor output, but rather via putative connections and correlations between several recording and stimulating electrodes. They found that functional connections between neurons in sensorimotor cortical networks can be changed and reorganized after persistent spike timing dependent stimulation [88]. Parallel to the electrical stimulation, optogenetic stimulation techniques have recently been tested as well. Specific neuronal types can be activated or inhibited, and thus opens up possibilities for modulating neural circuits with high levels of precision, for review see [89]. In essence, we can now further study what factors are involved in sensorimotor cortical synaptic plasticity in a variety of ways previously not possible. Rather than simply stimulating modulatory nuclei such as the ventral tegmental area or the nucleus bacillus we can record from these regions and pattern stimuli in the sensorimotor cortex in relation to their outputs, and so forth. Thus, a host of new possibilities opens up when using BMIs to probe neuroplasticity in the network.

### 2.3. Analysis Tools

Neural modulation of single cells during motor adaptation and motor learning is commonly analyzed by using peristimulus time histograms (PSTH), modulation depth, modulation strength and preferred direction, and so forth. While the power of the brain's computation lies strongly in its connections, to understand the function of neuronal circuits and systems, it is essential to characterize the connections between individual neurons [90, 91]. The cross-correlation, coherence, and joint peristimulus time histogram (JPSTH) methods have been used to test for coupling or correlation between pairs of cells in traditional neurophysiology [92, 93]. Besides the above commonly used methods, model-based methods have recently been used to characterize the strength and dynamics of putative connections between neurons. Granger causality is promising for continuous signals, and mutual information or transfer information for point processes, which have been used in computational neuroscience for analyzing spiking neural systems [94, 95]. 

However, plasticity can also be demonstrated at the network level, such as the map reorganization discussed previously. In many brain areas, each neuron receives input from a large population, with the advance of the large-scale simultaneous recording; new analysis methods have been proposed and strengthen our ability to understand these mechanisms. Generalized linear models (GLMs) provide a framework based on the point process representation of the spike trains. The GLM attempts to predict a neuron's activity based not only on its own activity and the activity of other neurons, but also on external inputs. By combining a Kolmogorov-Smirnov (KS) statistical analysis for the goodness of fit in each model, it can be expanded to neural networks and provides a powerful tool for neural network functional connectivity analysis [96–99]. The power of the GLM also lies in the fact that once the parameters have been estimated from the training dataset, the model can be used to predict the spiking activity of each neuron from testing dataset [99]. A new state space GLM is able to capture neural dynamics of individual cells at different time scales [100, 101]. To deal with a nonunique solutions of GLMs, a variety of techniques including the regularization method, Bayesian approach, calculation of the maximum a posteriori (MAP), and modulated poisson or renewal methods are used to estimate functional connectivity of spike train ensembles [102, 103].

**Figure 5:**
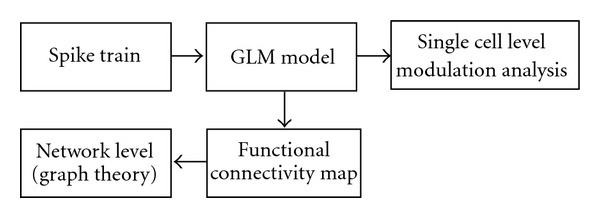
Sketch of the GLM-based plasticity analysis.

The procedures for analyzing neural modulation based on GLM models are shown in Figure 5. Graph theory can be applied to analyze the connection maps [104]. Although GLMs have been used to analyze neural dynamics from both individual cells and the functional connection in a network [97, 98, 100, 101], advanced analysis tools may be necessary for larger scale nonlinear neural dynamic analysis and modeling [90]. Understanding the patterns of neural network changes may be essential in order to provide a detailed picture of how the motor cortex is involved in normal motor adaptation and neural plasticity during motor learning. It is worth noting that the connections or connectivity maps constructed from these types of approaches only suggest connectivity and certainly do not prove it.

## 3. Conclusion

In this brief paper, we have discussed some of the recent neurophysiological and behavioral work over the past few decades that indicate the relevance synaptic plasticity plays in the sensorimotor cortex for sensorimotor learning in the RGR task. There is now a good deal of evidence that the sensorimotor cortices remain rather plastic throughout life, even if these brain regions seem more resistant to plasticity as compared to other areas such as the hippocampus. Within the sensorimotor cortex plasticity appears to be gated by modulatory systems such as the dopaminergic and cholinergic systems. In addition, learning occurs on at least two clear timescales, the short term, on the order of trials to days, where the learning curve is very steep, and on a longer timescale where learning is much slower. Associated with these two timescales may be changes to the physical substrate, such as synaptogenesis and remodeling of synaptic spines that may allow the system to undergo even further learning in the future. 

The advancement of simultaneous multiple electrode recordings, BMI technology, and optogenetics combined with our growing knowledge of the molecular machinery involved in synaptic plasticity, and new pharmacological agents capable of interfering with this machinery are providing new experimental tools to research sensorimotor plasticity and motor learning. In addition to new experimental tools, new mathematical methods may be necessary as well to allow us to fully elucidate how information processing and memory storage capacities of neural networks lead to changes in performance. With our increased understanding of neural reorganization, we could drive function-enabling plasticity and prevent function-disabling plasticity. Thus, this knowledge can be directed toward functional improvement and open up a new dimension in the care of the neurologically impaired patients. Our first push toward this goal involves the use of the PKM*ζ* inhibitor ZIP to alleviate focal hand dystonia and other neural plasticity-related disorders such as chronic pain, which has recently been alleviated via ZIP [105].
